# Certified Registered Nurse Anaesthetists’ and Critical Care Registered Nurses’ perception of knowledge/power in teamwork with Anaesthesiologists in Sweden: a mixed-method study

**DOI:** 10.1186/s12912-023-01677-z

**Published:** 2024-01-02

**Authors:** Jenny Wising, Madelene Ström, Jenny Hallgren, Komalsingh Rambaree

**Affiliations:** 1 School of Health Sciences, University of Skövde, Skövde, Sweden; 2 Region Västra Götaland, Skaraborgs Hospital Skövde, Dept of Anesthesia, Skövde, Sweden; 3 School of Health Sciences, University of Skövde, Skövde, Sweden; 4 Department of Social Work and Criminology, University of Gävle, Gävle, Sweden

**Keywords:** Anaesthesiologist, Nurse anaesthetist, CRNA, Critical care nurse, CCRN, Team

## Abstract

Efficient teamwork is crucial to provide optimal health care. This paper focuses on teamwork between Anaesthesiologists (ANES), Certified Registered Nurse Anaesthetists’ (CRNA) and Critical Care Registered Nurses (CCRN) working in challenging environments such as the intensive care unit (ICU) and the operating room (OR). Conflicts are common between physicians and nurses, negatively impacting teamwork. Social hierarchies based on professional status and power inequalities between nurses and physicians plays a vital role in influencing teamwork. Foucault was a famous thinker especially known for his reasoning regarding power/knowledge. A Foucauldian perspective was therefore incorporated into this paper and the overall aim was to explore CCRN/CRNA perception of knowledge/power in teamwork with ANES.

**Methods** A mixed-method approach was applied in this study. Data was collected using a web-based questionnaire containing both closed-end and open-ended questions. A total of 289 CCRNs and CRNAs completed the questionnaire. Data analysis was then conducted through five stages as outlined by Onwuebugzie and Teddlie; analysing quantitative data in SPSS 27.0 and qualitative data with a directed content analysis, finally merging data together in ATLAS.ti v.23.

**Results** The result reveals a dissonance between quantitative and qualitative data; quantitative data indicates a well-functioning interdisciplinary teamwork between CCRN/CRNA and ANES - qualitative data highlights that there are several barriers and inequalities between the two groups. Medicine was perceived as superior to nursing, which was reinforced by both social and organisational structures at the ICU and OR.

**Conclusion** Unconscious rules underlying current power structures in the ICU and OR works in favour of the ANES and biomedical paradigm, supporting medical knowledge. To achieve a more equal power distribution between CCRN/CRNAs and ANES, the structural hierarchies between nursing and medicine needs to be addressed. A more equal power balance between the two disciplines can improve teamwork and thereby reduce patient mortality and improve patient outcomes.

## Background

Team is more than a group of people who works together. According to Katzenbach and Smith [1], for a group to become a team it requires its members, who are often small in numbers, to have complementary skills, commitment to a common purpose, performance goals, and a common approach for which they hold themselves mutually accountable. Teams in organizations are crucial, as they have the potential for greater adaptability, productivity, and creativity than individuals can achieve alone [2]. In healthcare settings like intensive care units (ICU) and operating rooms (OR), interdisciplinary teamwork is vital for providing efficient care to critically ill patients in a challenging environment [3]. Well-established teamwork in these settings leads to lower patient mortality, improved outcomes, a productive work environment [4, 5] and has the potential to foster a more patient safe environment [6].

Interdisciplinary teams in the ICU and OR consists of different healthcare professionals, including Certified Registered Nurse Anaesthetists (CRNA), Critical Care Registered Nurses (CCRN) and Anaesthesiologists (ANES). The ANES have the greatest medical responsibility for anaesthesia and certain procedures in both settings [7]. The CRNA and CCRN take on leadership roles in nursing within the team, necessitating strong collaboration between the ANES, CCRN, and CRNA to provide high-quality care [8, 9]. In Sweden, ANES has a prominent medical role both in the ICU and the OR, working alongside both CCRNs and CRNAs, whereas CCRNs usually focus solely on the ICU and CRNAs predominantly operate in the OR. As of 2020, there was approximately 12,500 active CCRNs and CRNAs in Sweden, compared to around 2000 registered ANES [10].

Despite the crucial nature of teamwork, research findings have revealed significant challenges to collaboration between nurses and physicians in the ICU and OR [11–14]. Conflicts are common [12, 15, 16] and disrupt team collaboration, negatively impacting patients and families [16, 17]. Reasons for conflicts within the team include a lack of respect, inadequate knowledge sharing, and the presence of social hierarchies based on professional status and power dynamics [16]. Notably, status and power tend to interconnect and reinforce one another, as individuals with higher status within a hierarchy often gain more power, resulting in a further strengthening of their status. Power is not only used to solidify hierarchies but also leveraged to maintain or increase a person’s status within the hierarchy [18]. Furthermore, both power and hierarchies play vital roles in influencing teamwork [19–21].

To illuminate the potential broader structural issues and power dynamics within nurse- physician collaboration, this paper adopts a Foucauldian perspective. The conceptual and theoretical underpinnings of this study draw from Foucault’s insights into power/knowledge and are integrated with Katzenbach and Smith’s [1] conceptualization of a team. By combining these perspectives, our aim is to provide a comprehensive understanding of the dynamics at play in nurse-physician collaboration. The rationale behind incorporating an integrated conceptual/theoretical framework in this paper lies in its potential to enhance the interpretation of findings and facilitate a constructive discussion on teamwork in a structured manner [22]. Previous studies have employed a Foucauldian perspective for analyzing teamwork [23, 24]. Despite the widespread use of Foucault’s framework in research, its application in conjunction with a conceptual framework, such as Katzenbach and Smith’s definition of a team, appears to be non-existent (or rare). This article therefore brings a new layer of empirical evidence with an innovative integrated qualitative and quantitative data analysis for knowledge development in the field of teamwork among nursing staff members.

To further clarify the relevance of using a Foucauldian perspective, it is necessary to delve into the social positioning of medicine and nursing. Historically, medicine, and the male physician, have been perceived as superior to nursing, and the female nurse [19]. Recent research suggest that power inequalities still play a significant role in physician-nurse collaboration. This can be partially attributed to varying levels of education and professional status [25]. It is worth noting that this hierarchical status of medicine is deeply ingrained in both nursing and medical education [26]. Understanding the intricacies of this hierarchy becomes more crucial when adopting a Foucauldian perspective, which involves exploring the complex relationship between space, knowledge, and power [27].

According to Foucault, science is produced in an interaction of power relations that articulates knowledge, practices, and social relations; and scientific knowledge are therefore based on different epistemes - regimes of truth [28]. An episteme is a structure of social coding and ordering that organizes our consciousness, perception, and reflection and disposes us toward particular aspects of social phenomena [29] – such as power and hierarchies. Episteme is therefore condition of possibility of discourse under a particular period; it is an a priori set of rules of formation that allow discourses to function, that allow different objects and different themes to be spoken at one time but not at another [30]. For Foucault, each society (organization, institutions, relations etc.) has its own regimes of truth. In order to understand regimes of truth, one needs to study among others: the types of discourse (society) harbors and causes to function as true; the mechanisms and instances which enable one to distinguish true from false statements and the way in which each is sanctioned; the techniques and procedures which are valorized for obtaining truth; the status of those who are charged with saying what counts as true [31]. It is known that social structural hierarchies in healthcare is a potential harm towards interdisciplinary teamwork [12, 32]. Furthermore, these hierarchies based on the power dynamics between nursing and the biomedical paradigm may impede the implementation of nursing-based interventions, such as person-centered care [33].

Foucault furthermore describes power as diffused, embodied, and enacted in various entities rather than owned by individual or groups [34]. For Foucault, power comes from settings such as workplaces where subjects (like patients, nurses, and physicians) form power relations through discursive formations in daily interactions with others. In the discursive formation, Foucault sees discourses as contingent rules, beliefs, and practices that produce and organize knowledge and power to create subjects and the realities in which they live [35]. Foucault considered power and knowledge as closely intervened, writing it as “power/knowledge”, and argued that power is constituted through accepted forms of knowledge, stating that the power determines the production of knowledge [34]. The oxford dictionary defines knowledge as ‘the information, understanding and skills that you gain through education or experience’ [36]. Compared to the academic education of medicine dating back to the seventeenth century [37], nursing emerged as a relatively young academic discipline in Sweden in 1977 [38], while anesthesiology and intensive care, fairly new fields in medicine, made significant progress in Sweden during the mid-twentieth century [39]. Today in Sweden, the ANES requires a medical degree of 360 credits. After completing a basic six-month residency, the ANES undergo specialty training for at least five years [40]. The CCRNs and CRNAs are registered nurses who have completed a one-year master’s program (60 credits) with certification requirements, in addition to a bachelor’s degree in nursing (180 credits), specializing in either anesthesia or intensive care.

While the dynamics of teamwork in ICUs and ORs have been investigated before, societal developments and social reforms influence the dynamics of teamwork [21]. This necessitates continuous research to understand the current state of interdisciplinary teamwork. To our knowledge this is the only study exploring CCRN/CRNA perception of teamwork with ANES in Sweden, discussing the findings through a Foucauldian perspective using Katzenbach and Smiths definition of a team.

## Methods

The overall aim of this study was to explore CCRN/CRNAs perception of knowledge/power in teamwork with ANES in Sweden by answering the following research questions: (1) ‘how does power affect the CCRN/CRNA perception teamwork with the ANES?’ and (2) ‘how does knowledge affect CCRN/CRNA perception of teamwork with the ANES?’ The design was a convergent mixed method, where both quantitative and qualitative data was collected and analyzed at the same time. The main purpose of the convergent mixed method analysis was triangulation, so that quantitative and qualitative findings may be mutually corroborated [41]. The two forms of data are analyzed separately and then merged together in creating discussion based on the findings [42]. This study can therefore be categorized as being a partially mixed concurrent dominant model as per the typology of mixed method design presented by Leech and Onwuegbuzie [43]. The quantitative method had a dominant status in this study as a questionnaire was the instrument used for data collection. Creswell and Clark [44] argue that, although a questionnaire may not include a rich collection of qualitative data, it does meet the minimum criteria spelled out in the definition of mixed methods research.

### Sample and data collection

Participants were recruited using two methods: (1) hospitals in each of Sweden’s 21 regions were e-mailed the link to the online questionnaire, asking the unit manager for permission and, if it was granted, to forward the link to the CCRN/CRNA; (2) an open request was posted on three closed groups on a social media platform: one group exclusive for CCRNs, one group exclusive for CRNAs and one group exclusive for RNs. Sampling through social media can prove to be both cost- and time-efficient, enabling the targeted engagement of a specific group of respondents [45]. This was the reason why this method was included as an additional sampling strategy, combined with sending out the link by e-mail to unit managers. Data was collected during three weeks in October 2021. A total of 292 participants completed the questionnaire; three were excluded because they were not currently working in anaesthesia or intensive care which resulted in a total of 289 completed questionnaires including 343 open responses (considered here as qualitative data).

### Questionnaire

Data collection was carried out through a web-based questionnaire. A face validity questionnaire was created with questions based on Katzenbach and Smith’s definition of a team [1]. The questionnaire included 24 questions (Table 1), starting with demographic data (age, gender etc.). Statements were formulated according to a Likert scale ranging from 1 to 5, and multiple choice closed-ended questions were crafted regarding whether gender and the number of years in the profession affect the perception of teamwork. Furthermore, the questionnaire comprised three open-ended questions answered in the free text (qualitative data): (1) *“If you answered yes, do you wish to discuss more about when you were treated condescendingly by an ANES?”;* (2) *“If you think there is room for improvement in the collaboration between the ANES and the CRNA/CCRN, what would you wish to improve”*; (3*) “Do you want to add anything about the CRNA/CCRN’s collaboration with the ANES beyond what has been asked in this questionnaire?”.* Before distributing the questionnaire to the sample, five independent CCRN/CRNA executed a pilot trial resulting in no additional changes.

**Table 1:** Included statements in the questionnaire

Statements in Questionnaire	
Teamwork exists with the ANES^a^	You have avoided consulting the ANES due to their previous condescending behaviour^a^
Better cooperation with the ANES compared to your previous teamwork experience with physicians as a RN^a^	Improvement is needed within the team^a^
Rank the occupational categories according to what you consider to be the hierarchy of a care team^b^	Your skills are utilised by the ANES^a^
Communication between the CRNA/CCRN and the ANES works well^a^	The ANES is dependent on you for their work^a^
The ANES respects your competence ^a^	You depend on the ANES for your work^a^
You respect the ANES’ competence^a^	Your gender affects how much space and decision-making right the ANES offers you^d^
The ANES understands your profession^a^	The gender of the ANES affects how much space and decision-right you give them in the team^e^
You make shared decisions with the ANES ^a^	The gender of the ANES affects how much space and decision-making right the ANES offers you in the team^f^
The ANES appreciates you in the team^a^	Professional years you spent as a CRNA/CCRN affect how much space and decision-making right the ANES gives you in the team^g^
The ANES provides you opportunities to influence the care within the team ^a^	Professional years of the ANES affect how much space and decision-making right the ANES give you in the team^h^
You have been treated condescendingly by the ANES while working together ^c^	

^a^Likert scale 1–5: 1 = strongly agree, 5 = do not agree at all; ^b^the assistant nurses, the CRNA/CCRN and the ANES were paired with a number: 1 = highest in the hierarchy, 2 = in the middle and 3 = lowest in the hierarchy; ^c^Likert scale 1–5: 1 = never, 5 = always; ^d^multiple choice question: yes, male CRNA/CCRNs get more power; yes, female CRNA/CCRNs get more power; no difference; ^e^multiple choice question: yes, CRNA/CCRNs give more space to female ANES; yes, CRNA/CCRNs give more space to male ANES; no difference; ^f^multiple choice question: yes, male ANES give more space and decision-making right; yes, female ANES give more space and decision-making rights; no difference; ^g^multiple choice question: yes, the longer you work, the more space and decision-making right you get; yes, the shorter you work, the more space and decision-making right you get; no difference; ^h^multiple choice question: yes, the longer the ANES has worked, the more space and decision-making right is given to the CRNA/CCRNs; yes, the longer the ANES has worked, the less space and decision-making right is given to the CRNA/CCRNs; yes, the shorter the ANES has worked, the more space and decision-making right is given to the CRNA/CCRNs; yes, the shorter the ANES has worked, the less space and decision-making right is given to the CRNA/CCRNs; no difference

### Data analysis

**Fig. 1:**
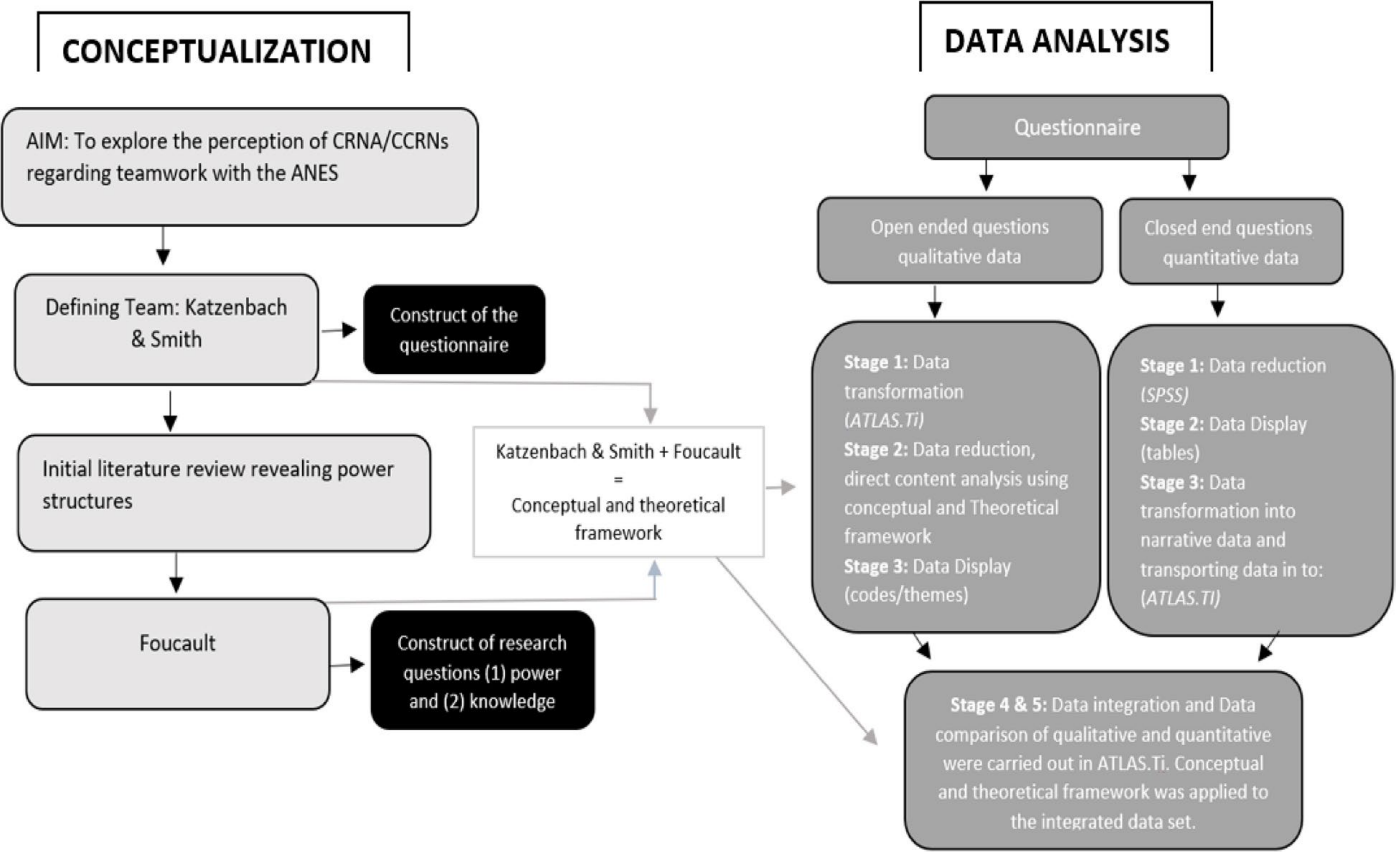
Flow chart of conceptualization and data analysis

The data analysis process in this study followed five out of the seven stages described by Onwuegbuzie and Teddlie [46]: data reduction, data display, data transformation, data intergration, and data comparison. However, the stages were not conducted in a stepwise manner regarding qualitative data, as the qualitative data analysis was performed using ATLAS.ti v.23 softare instead of manual methods. The use of ATLAS.ti required the transformation of qualitative data before reduction and display. A flow chart depicting the conceptation and data analysisprocess used in this paper, is provided in Fig. 1.

As it can be found from the flow chart, the research process followed a deductive approach through a literature review for developing a conceptual/theoretical framework. Katzenbach and Smith [1] was used for conceptualising – Team. Foucault’s theorisation on power/knowledge was used as a framework for structuring the analysis and discussion. Data analysis of quantitative data started with *data reduction,* meaning reducing the dimensionality of collected data [46]. *Data reduction* is a communication process used by researchers for reporting raw scientific research data into easily interpreted and revealing numerical, narrative, and visual descriptions that can help to make the research findings understandable to a broad range of audiences [47]. Quantitative data was analyzed with descriptive and analytical statistics using SPSS 27.0. T-test and ANOVA were used to compare the differences between groups. Univariate linear regression analyses were performed on the factors that influenced the existence of teamwork and whether the team needed improvement. The significant variables from the analyses were entered into a multivariate linear regression. The literature review for the background revealed that changes in history and gender influenced perceptions of CRNA/CCRNs on teamwork. Based on this assumption, correlations on gender, participants’ age and years in the profession were performed. A *p*-value > 0.05 was considered significant. The second step in analysing quantitative data was *data display,* and quantitative data was structured in tables (Tables 2, 3, 4, 5, 6 and 7) followed by the third step, *data transformation.* During this stage of analysis, quantitative data are converted into narrative data that can be analysed qualitatively [44]. In this process the survey data from SPPS 27.0 was imported to ATLAS.ti V.23.

**Table 2:** Demographic characteristics of participants

	All, n (%)	Age, mean (sd)	Years of experiences as specialist nurse, mean (sd)
All	298 (100)	44.1 (10.41)	11.6 (9.45)
Men	74 (25.6)	41.9 (10.44)	9.46 (8.32)
Women	215 (74.6)	44.77 (10.40)	12.29 (9.56)
CRNA	142 (49.0)	41.46 (9.42)	9.17 (8.23)
CCRN	114 (40.0)	46.09 (10.94)	12.80 (9.58)
CRNA and CCRN	33 (11.0)	49.24 (10.38)	19.93 (10.38)

*CRNA* Certified Registered Nurse Anaesthetist, *CCRN* Critical Care Registered Nurse

**Table 3:** Overview of the quantitative data from the questionnaire

	Total Mean (sd)	Men Mean (sd)	Women Mean (sd)	CRNA Mean (sd)	CCRN Mean (sd)	CRNA/CCRN Mean (sd)	
	*n = 289*	*n = 74*	*n = 215*	*n = 142 (49%)*	*n = 114 (40%0*	*n = 33 (11%)*	p-value*
Teamwork exist with the ANES	1.56 (0.83)	1.50 (0.80)	1.55 (0.79)	1.63 (0.86)	1.45 (0.66)	1.45 (0.87)	0.139
Better cooperation with ANES	1.72 (0.96)	1.78 (1.02)	1.70 (0.94)	1.74 (0.93)	1.72 (0.99)	1.67 (1.03)	0.927
Communication works well with ANES	1.94 (0.70)	1.86 (0.63)	1.97 (0.72)	1.96 (0.74)	1.92 (0.61)	1.88 (0.82)	0.775
ANES respects your knowledge	1.90 (0.82)	1.89 (0.82)	1.90 (0.82)	1.96 (0.88)	1.87 (0.73)	1.76 (0.79)	0.391
You respect the ANES competence	1.56 (0.62)	1.62 (0.57)	1.54 (0.63)	1.58 (0.63)	1.54 (0.60)	1.55 (0.62)	0.877
ANES understands your profession	1.93 (0.91)	2.05 (0.95)	1.89 (0.90)	1.91 (0.97)	1.95 (0.88)	1.94 (0.79)	0.941
Shared decisions with the ANES	1.92 (0.82)	1.85 (0.96)	1.94 (0.77)	1.96 (0.91)	1.88 (0.74)	1.89 (0.74)	0.712
ANES appreciates you in the team	1.83 (0.81)	1.79 (0.76)	1.84 (0.83)	1.84 (0.86)	1.82 (0.75)	1.79 (0.82)	0.954
The ANES provides you opportunity to influence the care within the team	1.83 (0.82)	1.75 (0.81)	1.85 (0.82)	1.82 (0.84)	1.82 (0.77)	1.88 (0.91)	0.944
You have been treated condescendingly by ANES while working together	2.30 (0.90)	2.32 (0.95)	2.29 (0.88)	2.29 (0.87)	2.31 (0.91)	2.30 (0.98)	0.983
You have avoided consulting with the ANES due to their condescending behaviour	2.11 (1.02)	1.99 (1.04)	2.15 (1.01)	2.10 (1.01)	2.16 (1.04)	1.97 (0.98)	0.683
Improvement is needed within the team	2.13 (1.08)	2.01 (1.08)	2.17 (1.08)	2.20 (1.08)	2.04 (1.11)	2.15 (1.00)	0.530
Your skills are utilized by the ANES	1.94 (0.89)	1.99 (0.93)	1.93 (0.87)	2.03 (0.96)	1.88 (0.79)	1.82 (0.85)	0.274
ANES are dependent on you in work	1.26 (0.59)	1.28 (0.63)	1.25 (0.58)	1.27 (0.68)	1.26 (0.53)	1.15 (0.36)	0.555
You depend on the ANES for your work	1.56 (0.83)	1.66 (0.95)	1.52 (0.78)	1.62 (0.93)	1.41 (0.66)	1.82 (0.81)	0.021
Your gender affects how much space and decision-making right the ANES offers you in the team							
*Men more power*	*n* = 47 (16.4%)	*n* = 6 (8.2%)	*n* = 41 (19.2%)	*n* = 22 (15.6%)	*n* = 17 (15%)	*n* = 8 (25%)	
*Women more power*	*n* = 2 (0.7%)	*n* = 1 (1.4%)	*n* = 1 (0.5%)	*n* = 11 (0.7%)	*n* = 0 (0.9%)	*n* = 0	
*No difference*	*n* = 237 (82.9%)	*n* = 66 (90.4%)	*n* = 171 (80.3%)	*n* = 118 (83.7%)	*n* = 95 (84.1%)	*n* = 24 (75%)	
The gender of the ANES affects how much space and decision-right you give them in the team							
*Women more power*	*n* = 1 (0.4%)	*n* = 0	*n* = 1 (0.5%)	*n* = 1 (0.7%)	*n* = 0	*n* = 0	
*Men more power*	*n* = 22 (7.9%)	*n* = 3 (4.2%)	*n* = 19 (9.1%)	*n* = 11 (7.9%)	*n* = 9 (8.2%)	*n* = 2 (6.5%)	
*No difference*	*n* = 257 (91.8%)	*n* = 68 (95.6%)	*n* = 189 (90.4%)	*n* = 127 (91.4%)	*n* = 101 (91.8%)	*n* = 29 (93.5%)	
The gender of the ANES affects how much space and decision-making right the ANES offers you in the team							
*Women more power*	*n* = 12 (4.2%)	*n* = 1 (1.4%)	*n* = 11 (5.2%)	*n* = 5 (3.5%)	*n* = 7 (6.1%)	*n* = 0	
*Men more power*	*n* = 20 (7%)	*n* = 7 (9.6%)	*n* = 13 (6.1%)	*n* = 13 (9.2%)	*n* = 4 (3.5%)	*n* = 3 (10%)	
*No difference*	*n* = 254 (88.8%)	*n* = 65 (89%)	*n* = 189 (88.7%)	*n* = 124 (87.3%)	*n* = 103 (90.4%)	*n* = 27 (90%)	
Professional years as a CRNA/CCRN affects how much space and decision-making right the ANES gives you in the team							
*Longer and more*	*n* = 241 (83.7%)	*n* = 63 (85.1%)	*n* = 178 (83.2%)	n = 124 (87.3%)	*n* = 90 (79.6%)	*n* = 27 (81.8%)	
*Shorter and more*	*n* = 3 (1%)	*n* = 1 (1.4%)	*n* = 2 (0.9%)	*n* = 2 (1.4%)	*n* = 1 (0.9%)	*n* = 0	
*No difference*	*n* = 44 (15.3%)	*n* = 10 (13.5%)	*n* = 34 (15.9%)	*n* = 16 (11.3%)	*n* = 22 (19.5%)	*n* = 6 (18.2%)	
Professional years of the ANES affects how much space and decision-making right the ANES give you in the team							
*Longer and more*	*n* = 179 (61.9%)	*n* = 48 (64.9%)	*n* = 131 (60.9%)	*n* = 96 (67.6%)	*n* = 60 (52.6%)	*n* = 23 (69.7%)	
*Longer and less*	*n* = 6 (2.1%)	*n* = 2 (2.7%)	*n* = 4 (1.9%)	*n* = 2 (1.4%)	*n* = 3 (2.6%)	*n* = 1 (3%)	
*Shorter and more*	*n* = 18 (6.2%)	*n* = 6 (8.1%)	*n* = 12 (5.6%)	*n* = 9 (6.3%)	*n* = 8 (7%)	*n* = 1 (3%)	
*Shorter and less*	*n* = 26 (9%)	*n* = 7 (9.5%)	*n* = 19 (8.8%)	*n* = 16 (11.3%)	*n* = 7 (6.1%)	*n* = 3 (9.1%)	
*No difference*	*n* = 60 (20.8%)	*n* = 11 (14.9%)	*n* = 49 (22.8%)	*n* = 19 (13.4%)	*n* = 36 (31.6%)	*n* = 5 (15.2%)

**Table 4:** Univariate linear regression factors influencing the existence of teamwork and need of improvements

	Teamwork exist with the ANES		Improvement is needed within the team	
	β	*p-value*	Β	*p-value*
Teamwork exists with the ANES			−0.259	0.001
Better cooperation with the ANES compared to your previous teamwork experience with physicians as a RN	0.248	< 0.001	−0.037	0.587
Communication between the CRNA/CCRN and the ANES works well	0.474	< 0.001	−0.408	< 0.001
Your competence is respected by the ANES	0.383	< 0.001	−0.391	< 0.001
You respect the ANES competence	0.491	< 0.001	−0.274	0.008
The ANES understands your profession	0.380	< 0.001	−0.391	< 0.001
You make joint decisions with the ANES	0.387	< 0.001	−0.278	< 0.001
The ANES appreciates you in the team	0.463	< 0.001	−0.383	< 0.001
The ANES provides you opportunity to influence the care within the team	0.398	< 0.001	−0.306	< 0.001
You have been treated condescendingly by the ANES while working together	0.125	0.015	−0.325	< 0.001
You have avoided consulting with the ANES due to their condescending behaviour	0.137	0.002	−0.264	< 0.001
Your skills are utilized by the ANES	0.336	< 0.001	− 0.341	< 0.001
The ANES is dependent on you for their work	0.272	< 0.001	0.041	0.702
You depend on the ANES for your work	0.201	< 0.001	−0.071	0.362
The gender of the ANES affects how much space and decision-making right the ANES offers you in the team^*^	0.586	< 0.001	−0.357	0.078
The gender of the ANES affects how much space and decision-right you give them in the team^*^	0.198	0.106	−0.137	0.417
Professional years as a CRNA/CCRN affects how much space and decision-making right the ANES gives you in the team ^*^	−0.098	0.235	0.239	0.175

^*^Dichotomized (Yes = 1; No = 0)

**Table 5:** Multivariate linear regression on factors signaling existence of teamwork between CCRN/CRNAs and ANES

	β	Standard error (β)	*p-value*
Better cooperation with the ANES compared to your previous teamwork experience with physicians as a RN	0.118	0.045	0.010*
Communication between the CRNA/CCRN and the ANES works well	0.188	0.081	0.021*
Your competence is respected by the ANES	−0.034	0.087	0.695
You respect the ANES’ competence	0.109	0.082	0.188
The ANES understands your profession	0.127	0.061	0.037*
You make joint decisions with the ANES	0.076	0.067	0.260
The ANES appreciates you in the team	0.135	0.088	0.125
The ANES provides you opportunity to influence the care within the team	−0.015	0.082	0.856
You have been treated condescendingly by the ANES while working together	−0.008	0.054	0.879
You have avoided consulting with the ANES due to their condescending behaviour	−0.053	0.049	0.277
Your skills are utilised by the ANES	0.013	0.075	0.864
The ANES is dependent on you for their work	0.080	0.072	0.267
You depend on the ANES for your work	0.088	0.052	0.093
The gender of the ANES affects how much space and decision-making right the ANES offers you in the team	0.254	1.131	0.054

* = significant

**Table 6:** Multivariate linear regression on factors signaling whether improvements are needed in a team

	β	Standard error (β)	*p-value*
Communication between the CRNA/CCRN and the ANES works well	−0.176	−0.112	0.122
Your competence is respected by the ANES	−0.006	− 0.005	0.961
You respect the ANES’ competence	0.071	0.039	0.556
The ANES understands your profession	−0.301	− 0.255	0.001*
You make joint decisions with the ANES	−0.009	−0.007	0.933
The ANES appreciates you in the team	−0.137	−0.101	0.294
The ANES provides opportunities for you to influence the care within the team	0.031	0.023	0.793
You have been treated condescendingly by the ANES within the teamwork	−0.210	− 0.173	0.008*
You have avoided consulting with the ANES due to their condescending behaviour	−0.065	−0.061	0.362
Your skills are utilised by the ANES	0.068	0.056	0.530

* = significant

**Table 7:** Correlations and T-test on gender, age and professional years

Domains	Sex^a^ *p*-value	Age^b^ *p*-value	Professional years^c^ *p*-value
You respect the ANES’ competence	0,387	0,055 r = 0,113	0,006* r = 0,162
The ANES understands your profession	0,387	0,093 r = 0,099	0,036* r = 124
You have avoided consulting with the ANES due to their condescending behaviour	0,232	0,044* r = 0,199	0,769 r = 0,017
The ANES’ gender affects how much space and decision-making right they offer you in the team	0,664	0,012* r = 0,148	0,358 r = 0,055
Better cooperation with the ANES compared to your previous teamwork experience with physicians as a RN	0,576	0,034* r = 0,129	0,076 r = 0,108
Your gender affects how much space and decision-making right the ANES offers you	0,014* F = (m 2.61; sd 0.79) M = (m 2.82; sd 0.56)	0,186 r = 0,054	0,360 r = 0,054

^a^T-test, ^b^Pearson Correlation, *ANES* Anaesthesiologist, *CRNA* Certified Registered Nurse Anaesthesia, *CCRN* Critical Care, *RN* Registered Nurse, *GP* General Practitioner, *F* Female, *M* Male, *m* mean, *sd* standard deviation, * = significant

Qualitative data analysis started with *data transformation,* following the steps as outlined in the ATLAS.ti v.23 manual (ATLAS.TI, 2023). In other words, the open-questions from the survey data were coded to be analysed using the ATLAS.ti software.^1^ Then after, the qualitative data from the open-ended survey questions were reduced (*data reduction)*. Qualitative data were analysed in accordance with Hsieh and Shannon’s [48] description of a directed content analysis using the ATLAS.ti software. For qualitative *data reduction*, open coding method was applied, at a first stage. The open-ended questions were read several times by the authors to undertake open coding focusing on how the answers from the respondents correlated to open codes such as trust, personality, communication. At a second stage, the narrative text summarizing the most important findings were then regrouped into higher level of data reduction that is commonly referred as theme in qualitative methodology. Here, a directed approach where analysis started with a conceptual/theoretical framework based on Katzenbach and Smith’s [1] conceptualisation of a team and Foucault’s theorisation on power and knowledge was used as guidance. That is, the first level of codes was linked and/or merged under higher level of corresponding codes that are related to the concepts identified in the literature review on Katzenbach and Smith’s [1] conceptualisation of a team and Foucault’s theorisation on power and knowledge. The *data display stage*, was carried out by structuring qualitative data in a list of codes/themes and network formats to make the result more reviewable.

**Fig. 2:**
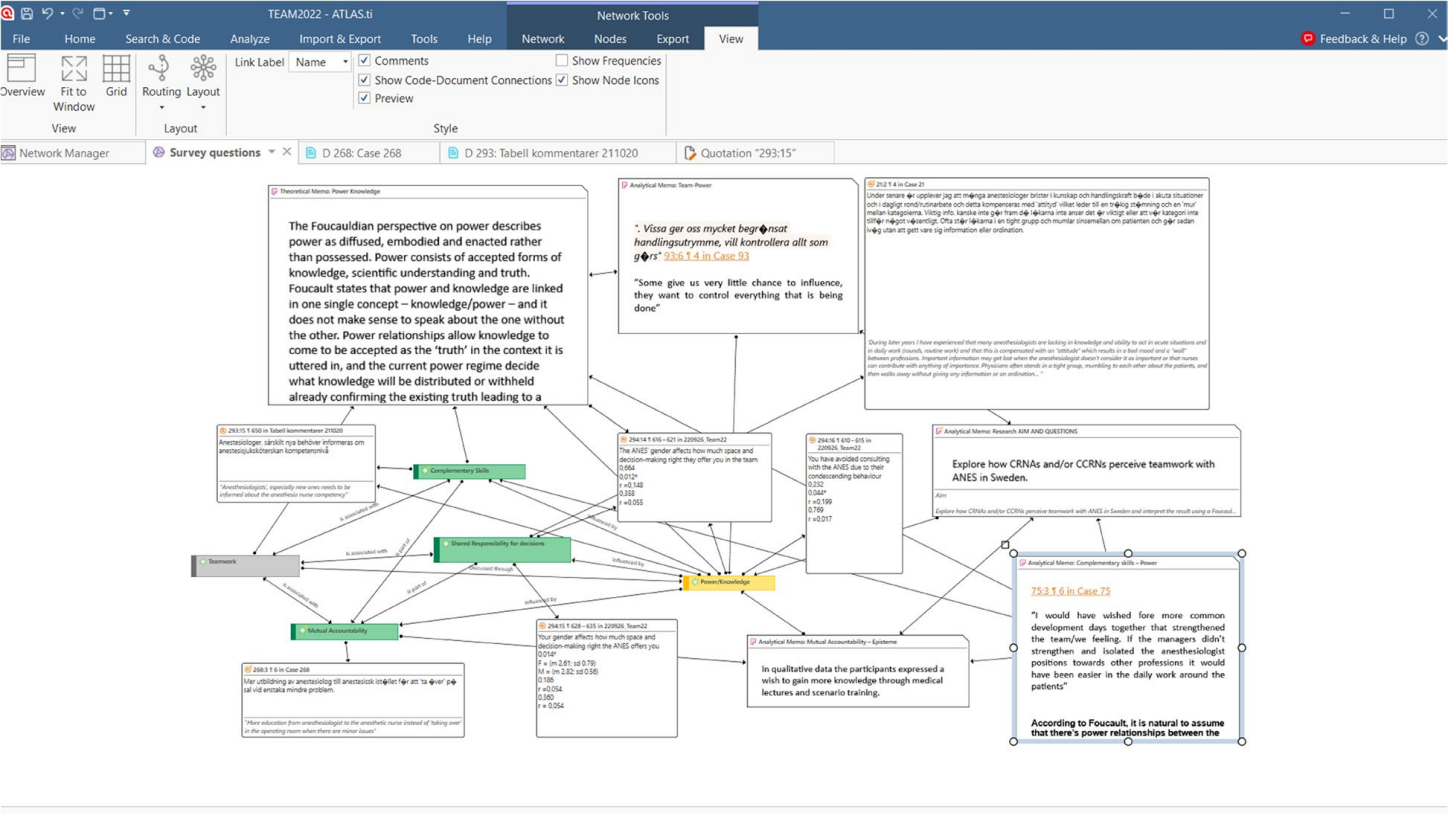
An example of a Network diagram for data analysis using ATLAS.ti software

Finally, *data integration* and *data comparison,* of qualitative and quantitative data was carried out. During this final stage qualitative and quantitative data was integrated as a coherent whole using ATLAS.ti. An example of data integration and data comparison is show in Fig. 2. A deductive approach using a conceptual/theoretical framework based on Katzenbach and Smith’s [1] conceptualization of teamwork and Foucault’s explanatory model regarding knowledge/power was applied to explore and compare quantitative and qualitive data.

## Results

In total, 289 questionnaires were returned, representing all 21 regions in Sweden. A majority of the participants were women, the distribution between CRNA and CCRN were, however, fairly even (Table 2).

### Quantitative data

Compared to CRNA and CCRN, the participants with both specialties perceived to a higher extent that they are less dependent on the ANES in their work, and an overview of the quantitative data is displayed in Table 3.

Analysing the factors affecting team improvements, the univariate linear regression revealed that communication, respecting and understanding others’ skills and professions, shared decision-making in a team, whether the ANES appreciates and offers opportunities for the CRNA/CCRN to influence the care, whether their skills were utilised, whether the ANES behaved condescendingly while working together and whether the CRNA/ CCRN thus avoided further consulting with the ANES were significantly associated (Table 4).

After entering the significant variables from the univariate linear regression into a multilevel regression, the ANES’ understanding of the CRNA/CCRN professions (*p* = 0.037), communication working well (*p* = 0.021) and better cooperation with the ANES (0.010) were significantly associated with the perception of existing teamwork (Table 5). The multivariate regression on the factors affecting whether improvements are needed in the team revealed significant associations with the ANES’ understanding of the CRNA/CCRN profession (*p* = 0.001) and whether the ANES behaved condescendingly during teamwork (*p* = 0.008) (Table 6).

The correlations revealed that younger CRNA/CCRN were more likely to avoid consulting an ANES due to the ANES’ past behaviour. The participants’ age also showed significant correlations with how much decision-right the ANES gave the CRNA/CCRNs and with the perception of better teamwork between ANES and CRNA/CCRN than between the RNs and physicians. The CRNA/CCRNs number of professional years also showed significant correlations with the participants’ respect for the ANES and the perception about whether the ANES knew about the CRNA/CCRNs profession: the higher number of professional years of the CRNA/CCRNs, the less respect they had for the ANES and the lower the perception of the ANES understanding of the CRNA/CCRNs profession. Moreover, gender revealed one significant correlation: the female participants perceived that their male colleges were given more decision-rights by the ANES (Table 7).

### Qualitative data

**Fig. 3:**
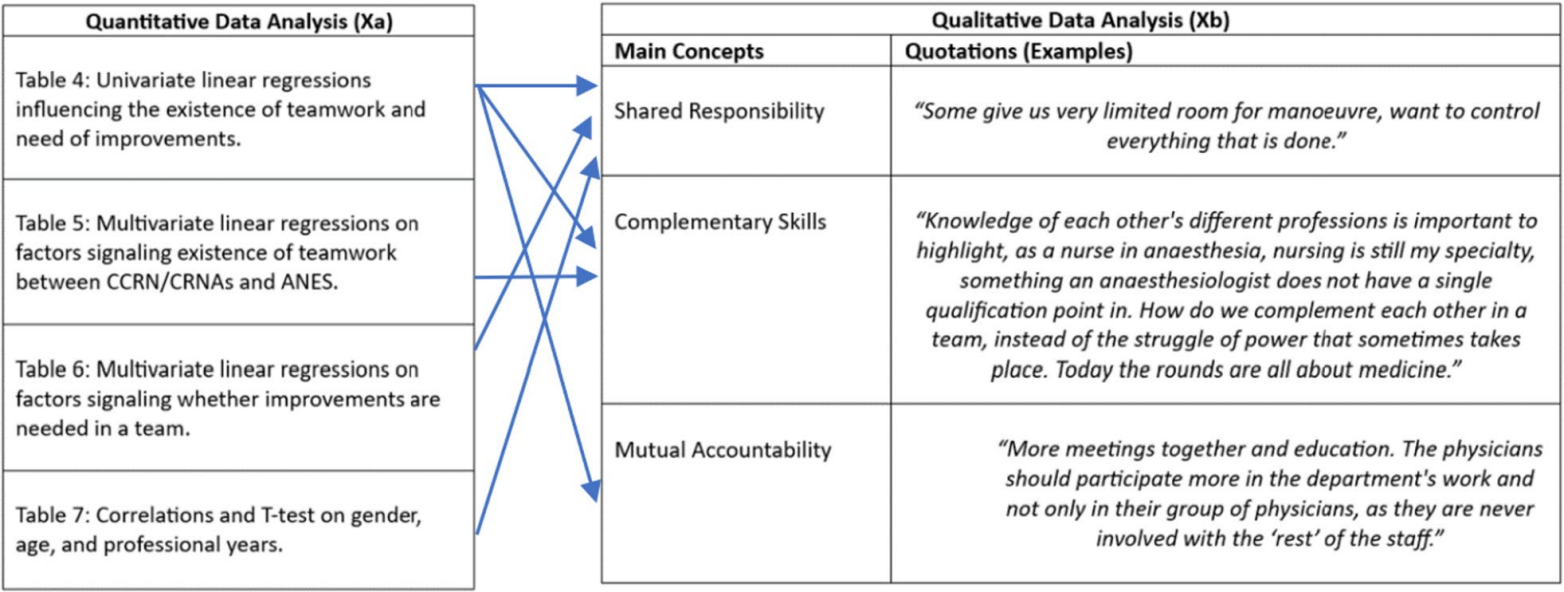
Integration of the quantitative and qualitative data in the analysis process

In the qualitative analysis, several themes based on the theoretical/conceptual network were constructed and explored. However, the findings presented in this section only focuses on three main ones that (a) answer the set research questions, and (b) allows deep exploration(qualitatively) of the main significant quantitative findings. Such an approach also allows for having more focused and in-depth discussion of the main findings using the Foucauldian perspective on power/knowledge affecting teamwork. The main qualitative themes (integrated with the quantitative data) that are presented are: shared responsibility for decisions, complementary skills, and mutual accountability. The tables (Xa and Xb) in Fig. 3 below show an integration of the quantitative and qualitative data in the analysis process based on Fig. 2 and Tables 4-7.

### Shared responsibility for decisions

Whereas the quantitative data showed that shared decision-making was significantly associated with team improvements, the qualitative data revealed that the participants were not trusted by the ANES, which counteracted the CRNA/CCRNs ability and participation in decisions In quantitative analysis, it became evident that a deficiency in the understanding of the CRNA/CCRNs profession by the ANES, together with condescending treatment, highlighted the necessity for improvements within the team. The qualitative data further revealed that the participants perceived the ANES as seeking control, displaying hierarchical power, which partially hampered trust and shared decision making expressed by one CCRN as following:

*“Some give us very limited room for manoeuvre, want to control everything that is done.”*

Quantitative data did not highlight gender as particularly significant. In qualitative data participants believed that the degree of space and decision-making rights are dependent on the ANES’ personality, and found it difficult to generalise this based on gender:

*“These are often characters that are not interested in teamwork or believe that the medical profession is the most important.”*

### Complementary skills

In quantitative data it became apparent that the utilizing of skills was significantly associated with factors affecting team improvement, and that CCRN/CRNAs to some extent perceived that the ANES lacked knowledge about the CCRN/CRNAs profession. In qualitative data the participants painted a nuanced picture of complementary skills, and many participants mentioned feeling their medical knowledge being underestimated by the ANES. A bullying attitude towards the CRNA/CCRNs was described: one participant referred to when an ANES said that “you are only a nurse”. A lack of respect from the ANES was also perceived, both for the nursing profession but also for the CRNA/CCRNs knowledge. The participants also felt deprived of the tasks they felt comfortable with, and thus perceived themselves as being undermined by the ANES.

The participants, furthermore, perceived that the discipline of nursing was less prioritised in favour of medicine; they wished that the two complemented each other more. Moreover, they wanted soft values such as emotions and experiences to be more discussed and the ANES to gain an increased understanding of nursing. This was expressed by one CCRN as following:

*“Knowledge of each other’s different professions is important to highlight, as a nurse in anaesthesia, nursing is still my specialty, something an anaesthesiologist does not have a single qualification point in. How do we complement each other in a team, instead of the struggle of power that sometimes takes place. Today the rounds are all about medicine.”*

### Mutual accountability

Quantitative data showed that better cooperation with the ANES were significantly associated with the perception of existing teamwork, which was also apparent in qualitative data. The participants wished for more shared activities: scenario trainings, lectures and so on, with the ANES. Above all, medical lectures and medical training were mentioned. However, some participants stated that the CRNA/CCRNs should give lectures on nursing to the ANES because in their current workplace it was only the ANES giving lectures to the CRNA/CCRNs, interpreted by the participants as the knowledge of nursing not being as important as the knowledge of medicine. Moreover, the participants also wanted the ANES to be more accessible in the ICU/OR and to offer learning opportunities when patient cases are discussed, which would increase the CRNA/CCRNs understanding of the situation:

*“More meetings together and education. The physicians should participate more in the department’s work and not only in their group of physicians, as they are never involved with the ‘rest’ of the staff.”*

## Discussion

The aim of this study was to explore the perception of the CRNA/CCRNs regarding teamwork with the ANES, focusing on how (a) power and (b) knowledge affected the perception, using a theoretical/conceptual network consisting of Katzenbach and Smiths description of a team and Foucault’s view of power/knowledge*.* Quantitative data revealed that the majority of the participants perceived that the CRNA/CCRN worked in a team with ANES. Qualitative data contributed with a partly contradictory description in comparison to quantitative data, and the final result reveals both perceived facilitators and barriers within teamwork in the ICU and OR.

### Power

Quantitative data revealed that the majority expressed a shared responsibility for decisions with the ANES, whereas qualitative data revealed that the CRNA/CCRN perceived being controlled by the ANES. This is not unique, as previous research have described the perception of working in teams even when teamwork was lacking [49]. This is interpreted as the participants not instinctively being aware of power structures. Foucault conceptualizes power in different forms, describing disciplinary power as a power that shapes and normalises subjects who eventually think and act in similar manners, and therefore facilitates appropriate behaviour [34]. Teamwork is a well-integrated concept in health care and nursing education, the appropriate behaviour is thus for the CRNA/CCRNs to respond positively to teamwork, which becomes clear in the quantitative data. Answering open-ended questions demands deeper reflection, the complexities concerning the participants’ perception of teamwork become more prominent. A more equal power balance between nursing and medicine would reduce structural hierarchies and strengthen communication and relationships in the team [50], improving interdisciplinary teamwork and therefore reduce patient mortality and improve patient outcomes [4, 5]. In order to achieve a balance in power, however, an awareness of existing structures must be established and highlighted among ANES and CRNA/CCRN.

Shared decision making was significantly associated with the perception if a team existed or not, and the ANES behaving condescendingly towards CRNA/CCRN was significant whether improvements where needed in a team. Qualitative data revealed that shared decision making was dependent on the ANES personality. Qualitative and quantitative data furthermore showed, unexpectedly, that gender had little impact on the perception of teamwork and shared decision making, thus challenging existing conceptions. There was one significant correlation: female CRNA/CCRNs perceived their male counterparts to be preferred by the ANES, in accordance with previous research [51]. Furthermore, the older the CRNA/CCRN, the likelier the participant perceived the ANES gender impacted teamwork. Foucault states that the truth is a product of the knowledge the power produces, and the episteme sets the structure for what will be considered as a truth. The ruling truth is then withheld by the society that follows and reproduces power relationships, obeying unconscious structures and the current episteme [29]. Society have considered female nurses subordinate to male physicians [19]. Social reforms, such as appointing more female physicians may change the system’s view on patriarchal power. As Foucault explain, power is always changing, and as the system changes, so will the truth and the episteme [34]. However, it is important, to acknowledge that female ANES reports gender bias in their workplace [52] and there’s an evident gender divide within the health workforce [53], thus even though this study doesn’t emphasize the impact of gender it is still an important matter to keep discussing. In the future male physicians as superior to female nurses may become an obsolete truth due to social reforms.

### Knowledge

Quantitative data revealed the perception that the ANES does not comprehend the CRNA/CCRNs profession, one of the most prominent finds in this study; affecting both if teamwork existed and if improvements where needed in the team. Qualitative data described medical knowledge as regarded superior to knowledge about nursing, describing both structural and social inequalities between the disciplines, comparing themselves with the ANES regarding medical knowledge in accordance with earlier research [54]. The result furthermore reveals that current power structures promoting the knowledge of medicine in ICUs and ORs make it difficult for CRNA/CCRNs to evolve within their own discipline of nursing in a clinical setting. For an example a participant expressed a lack of interdisciplinary discussions involving softer values such as expectations and emotions often represented by the nursing discipline. This is in accordance with earlier research stating that CCRNs vastly structure their work around objectified measures [55]. Foucault describes how physicians use the clinical gaze, meaning seeing the patient as an object rather than an individual [30], which is being premiered among nurses in a clinical setting [55], and is conflicting with nursing’s aim to see the person, and deliver a person-centred care which is one of nursing’s core competencies [8, 9]. The perceived superiority of medical knowledge is therefore a barrier towards the use of complementary skills, thus the ANES does not even know the complexity of the nursing profession, and not utilizing complementary skills is a common mistake in a team [1]. It is therefore important to highlight this matter, making sure that nursing as a skill and knowledge has an equal, obvious place within the team in relation to medicine.

One important finding is that a higher academic degree and increased knowledge– partly within medicine – significantly improved the CRNA/CCRNs perception of teamwork with the ANES, thus the participants experienced improved teamwork as a CRNA/CCRN with the ANES compared to being a RN in a team with a physician. In qualitative data the participants expressed a wish for more joint activities with the ANES, including lectures and scenario training. The solution to reinforce nursing academically to overcome interdisciplinary barriers was suggested by The Institute of Medicine [56], and received strong criticism from the American College of Physicians illuminating structural and territorial concerns between the two disciplines [57]. According to Foucault unconscious power structures are underlying the production of knowledge and science – and production of new knowledge may lead to a shift in power [34], which may explain why the American College of Physicians opposed the proposal. Mutual accountability is dependent on trust and commitments’ [1], and by the CRNA/CCRN increasing their knowledge within the current episteme in a clinical setting, biomedicine, the mutual accountability is argued to have been improved. It is therefore important for clinics to provide nurses with continuing education in order to strengthen teamwork.

### Limitations

This study contains several limitations which needs to be addressed. First this study’s method and design may be looked upon as a limitation. Although mixed-method is not a new method it is still lacking a common practice of how to combine quantitative and qualitative data in one study. A well-executed mixed method does, however, offer the possibility to combine the advantages in both qualitative and quantitative research resulting in a more profound understanding of the researched area [58]. To strengthen this study, the authors performed the data analysis by following five steps of conducting a mixed method described by Onwuegbuzie and Teddlie [46]. The decision to apply a theoretical/conceptual network might have narrowed the insights which can be considered a limitation. This approach did, however, help the authors to keep the consistency throughout the process – making clear connections between the empirical and theoretical foundation. Furthermore, the decision to early in the process create a network connecting Foucault with Katzenbach and Smith’s definition of team may have further narrowed the papers discussion. Considering the study’s complex aim and the large quantity of data collected the theoretical framework contributed to a structured interpretation of the result and is therefore argued to have enhanced this study’s inference quality.

Second, the questionnaire was pretested on a small group, and the face validity could be questioned. The questionnaire did, however, contain both numeric questions and open-ended questions which is considered a strength - by including open-ended questions in a questionnaire the data will more accurately reflect the participants conceptions, and therefore increase the study’s legitimation [59]. Third, the sampling was partly through social media which includes weaknesses such as a difficulty in following up and decreased control of whom actually participated, and it is important to acknowledge the notable limitation posed by the inability to calculate and report dropout rates. The absence of dropout rate data may hinder a comprehensive understanding of participant engagement and potential impacts on the study’s outcomes [60]. The recruitment strategy by social media can, however, also be considered as a strength by being an effective route to reach the targeted population and therefore improving response rate and increased confidentiality for participants [45]. In this study it was impossible to determine how many participants received the link via email from the unit manager and how many participants answered it through distribution via social media.

Fourth, limitations involve this study’s inference transferability, including the qualitative concept of transferability and the quantitative concept of external validity. External validity in quantitative research refers to the extent a result can be applicable to the wider population, whereas qualitative research often consists of a smaller sample and emphasise knowledge that is dependent on the context [58]. All participants who answered the questionnaire did not answer the open-ended questions, which might have resulted in that only the participants who were discontented with teamwork answered, leading to skewed qualitative data – a majority of the participants did however answer the open-ended questions. A strength in this study’s inference transferability is the large sample combined with participants from all 21 regions in Sweden, even though the distribution was too uneven to make any generalisations between the regions.

## Conclusion

Unconscious rules underlying current power structures works in favour of the ANES and biomedical paradigm, both regarding power and knowledge. In order to achieve a more equal power distribution between CCRN/CRNAs and ANES the structural hierarchies between nursing and medicine needs to be addressed within the team, thus a more equal power balance between the two disciplines can improve teamwork and thereby reduce patient mortality and improve patient outcomes. This study’s finding did not emphasize gender as a power factor, in contrast to earlier research, showing that in the future the male physician superior to the female nurse may become an obsolete truth. The perceived superiority of medical knowledge did, however, act as a hindrance towards the utilization of complementary skills within the team. Furthermore, increased formal knowledge improved the CCRN/CRNAs perception of working in a team, and it is therefore important to provide nurses with continuing education to improve teamwork. Further research, preferably including the perspective of the ANES, is needed in order to deepen the knowledge in the area.

## Data Availability

The datasets are available from the corresponding author on request.

## Abbreviations

CCRN: Critical Care Registered Nurse
CRNA: Certified Registered Nurse Anaesthetists
ANES: Anaesthesiologist
ICU: Intensive Care Unit
OR: Operating Room

## Footnotes

1 For more information, please read https://doc.atlasti.com/ManualWin/Documents/DocumentTypeSurveyImport.html
